# Comparison of objective visual quality between SMILE and FS-LASIK in moderate-to-high myopia

**DOI:** 10.3389/fmed.2024.1408516

**Published:** 2024-09-13

**Authors:** Huaxian Zou, Xianxian Wei, Lili Li, Diefeng Wei, Hejuan Mao, Yanyan Huang, Pengfei Lu, Ziyu Li, Dedong Zhong, Qi Chen

**Affiliations:** ^1^Visual Science and Optometry Center, The People's Hospital of Guangxi Zhuang Autonomous Region, Guangxi Key Laboratory of Eye Health, Guangxi Health Commission Key Laboratory of Ophthalmology and Related Systemic Diseases Artificial Intelligence Screening Technology, Institute of Ophthalmic Diseases, Guangxi Academy of Medical Sciences, Nanning, China; ^2^Graduate School of Guilin Medical University, Guilin, China

**Keywords:** SMILE, FS-LASIK, moderate-to-high myopia, corneal aberrations, optical quality

## Abstract

**Purpose:**

This study aims to compare the changes in the corneal wavefront aberrations and the objective visual quality resulting from two types of eye surgery—small incision lenticule extraction (SMILE) and femtosecond laser-assisted *in situ* keratomileusis (FS-LASIK)—in patients with moderate-to-high myopia.

**Methods:**

A prospective analysis was performed on 98 eyes of 51 patients who underwent SMILE. Additionally, 88 eyes of 45 patients who underwent FS-LASIK were analyzed. All patients underwent ocular examination preoperatively and at 1 day, 1 week, 1 month, and 3 months postoperatively. Corneal aberrations and objective visual quality were measured using the Optical Quality Analysis System II (OQAS II) and Optical Path Difference Scan III (OPD-Scan III).

**Results:**

At postoperative 1 day and 1 week, there was a statistically significant difference in uncorrected distance visual acuity (UDVA) between SMILE and FS-LASIK (*P* < 0.05). Postoperative spherical (S), cylinder (C) and spherical equivalent refraction (SE) were similar between the two groups (*P* > 0.05). In both groups, the absolute magnitude of total higher-aberrations (tHOA), piston, vertical tilt, vertical coma, and spherical aberration (SA) increased after surgery compared to preoperative values (*P* < 0.05). There was no significant difference in Δhorizontal tHOA, Δhorizontal tilt, Δhorizontal coma, and Δhorizontal trefoil between the two groups (*P* > 0.05), and the FS-LASIK had higher Δvertical trefoil and ΔSA (*P* < 0.05) but lower Δpiston, Δvertical tilt, and Δvertical coma than the SMILE group (*P* < 0.05). There was a rise in objective scattering index (OSI) and a decline in both modulation transfer function (MTF) cutoff and Strehl ratio (SR) after surgery compared to preoperative values in both groups (*P* < 0.05). There was a statistically significant difference in the OSI at 1 day and 3 months between the two groups (*P* < 0.05). Postoperative MTF cutoff and SR were similar between the two groups (*P* > 0.05). Postoperative OSI was positively correlated with corneal tHOA (0.261 ≤ *R* ≤ 0.483, *P* < 0.05) and was negatively correlated with vertical tilt and vertical coma (−0.315 ≤ *R* ≤ −0.209, *P* < 0.05) in both groups.

**Conclusion:**

While both SMILE and FS-LASIK can effectively correct moderate-to-high myopia, there is an increase in corneal aberrations and a postoperative delay in objective visual quality. The cornea may require a longer recovery period in the SMILE. OPD-Scan III combined with OQAS II is a useful supplementary inspection for assessing the optical quality following refractive surgery.

## Introduction

Small incision lenticule extraction (SMILE) and femtosecond laser-assisted laser *in situ* keratomileusis (FS-LASIK) are currently the more popular laser surgical procedures for correcting myopia/myopic astigmatism (1, 2). Both procedures are based on laser technology and are characterized by short treatment times, small focusing spaces, high precision, and low damage to surrounding tissues and organs (3, 4). SMILE has demonstrated its potential benefits of reduced denervation, no flap-related risks, and faster resolution of postoperative dry eye (5–7). Several studies have shown that irregular changes in corneal morphology and increased corneal higher-order aberrations after refractive surgery are the main reasons for the reduced optical quality after surgery (8, 9). Previous studies have found that although corneal higher-order aberrations are higher in FS-LASIK than in SMILE, better uncorrected distance visual acuity (UDVA) is achieved earlier after FS-LASIK (10–12). However, the correlation between higher-order aberrations and visual quality has not been conclusively established (13, 14).

In recent years, different types of aberrometers have been widely used in corneal refractive surgery (15–17). The Optical Path Difference Scan III (OPD-Scan III) is a five-in-one device including a topographer, keratometer, pupillometer, refractometer, and wavefront aberrometer (18). The device can simultaneously measure the total, cornea, and intraocular zeroth-eighth order wavefront aberrations. The zeroth-order aberration is a piston and represents refractive media clouding, which can further quantify corneal recovery after refractive surgery. The first-order aberration represents corneal tilt, quantifies the effect of intraoperative cutting on corneal morphology, and may be of significance in evaluating the correlation between postoperative corneal astigmatism and off-center cutting. The device is rarely used to evaluate optical quality in corneal refractive surgery. The Optical Quality Analysis System II (OQAS II), employing the double-pass technique, is recognized for its ability to quantitatively measure ocular scatter (19, 20). It is the world's leading optical device for objective, quantitative, comprehensive, and accurate analysis of visual quality. OQAS II and OPD-Scan III analyzers may have some clinical value in postoperative optical quality assessment.

Since there are still no consistent conclusions on the comparative results, we designed this study and mainly used OPD-Scan III and OQAS II as visual quality inspection equipment to compare and analyze the corneal aberrations and objective visual quality of patients who underwent SMILE and FS-LASIK for correcting moderate-to-high myopia in the early postoperative period.

## Methods

### Patients

This was a prospective cohort study. A total of 96 patients (186 eyes) with moderate-to-high myopia who underwent SMILE and FS-LASIK at the Visual Science and Optometry Center of the People's Hospital of Guangxi Zhuang Autonomous Region between January 2021 and June 2022 were enrolled. This study was approved by the Ethics Committee of the People's Hospital of Guangxi Zhuang Autonomous Region (No. IIT-2023-95) and complied with the Declaration of Helsinki (as revised in 2013). Informed written consent was obtained from all participants.

### Recruitment criteria

The inclusion criteria were as follows: (1) preoperative age between 18 and 35 years; (2) The manifest refractive spherical equivalent was −3.25D to −10.00D and the cylinder was from 0D to −2.00D; (3) Corrected distance visual acuity (CDVA) ≥1.0; (4) A stable refractive status for at least 2 years; (5) A requirement to discontinue the use of rigid contact lenses for at least 1 month and soft contact lenses for a minimum of 2 weeks; (6) Absence of dry eyes and any systemic or ocular disease; and (7) Central corneal thickness (CCT) ≥ 480 nm and residual stromal thickness (RST) ≥ 280 nm.

### Surgical procedure

The same experienced surgeon conducted all procedures. SMILE was conducted using the VisuMax 500 laser system (Carl Zeiss Meditec AG, Jena, Germany). The laser had a repetition rate of 500 kHz and a pulse energy of 130 nJ. The lenticule diameter was set between 6.0 mm and 6.5 mm, the cap diameter was between 7.0 mm and 7.5 mm, and the corneal cap thickness ranged from 110 μ to 120 μ. Spiral cuts were made on the anterior and posterior surfaces. The incision was positioned at 120° and had a width of 2.0 mm.

In the FS-LASIK, the cornea flap was created using the Intralase IFS 150 femtosecond Laser (Abbott Medical Optics, AMO Manufacturing, USA). The repetition rate of the laser was 150 kHz, and the pulse energy was 135–150 nJ. The intended flap thickness was 100 μm, resulting in a flap with a diameter ranging from 8.7 mm to 8.9 mm and a superior hinge position. After all the bubbles were absorbed under the corneal flap, the flap was lifted, and the excimer laser cutting was performed with a combination of small and large spots using an excimer laser machine [VISX Star S4 Excimer Laser System (Advanced Medical Optics Inc, Santa Clara, CA, USA)]. The cutting light zone was 6.0–6.5 mm, the transition zone was 0.5 mm, and the flap was reset after the rinsing postoperative flap and stroma with a balanced salt solution.

The treatment regimen was identical for both groups after the surgery. It included applying tobramycin dexamethasone (Alcon Couvreur, Belgium) four times a day during the initial week, followed by using 0.1% fluorometholone (Santen, Japan) four times a day for the following 3 weeks, and then using 0.1% hyaluronic acid sodium (Ursapharm Arzneimittel GmbH, Germany) and The deproteinized calf blood extract eye gel (5g, 20%, Shenyang Xingqi Pharmaceutical Co. Ltd., China) four times a day for a month.

### Preoperative and postoperative assessment

Each examination was performed by the same operator. Each patient received a comprehensive ophthalmological examination before the procedure. All inspections were performed in a dark room. The wavefront analysis system (OPD-Scan III, Nidek, Japan), utilizing a 6.0 mm pupil diameter, was employed to obtain the measurements of the corneal higher-aberrations (HOAs), including total higher-aberrations (tHOAs), piston, tilt (vertical tilt, horizontal tilt), trefoil (vertical trefoil, horizontal trefoil), coma (vertical coma, horizontal coma), and spherical aberration (SA). The OQAS II analyzer (Vision Metrics, Spain) was used to measure the MTF cutoff, objective scattering index (OSI), and Strehl ratio (SR). During these measurements, the patient's refractive error was entered into the system and fully corrected. Objective refraction was first measured, followed by an evaluation of the MTF cutoff, OSI, and SR. The measurements, which included visual acuity, objective refraction, corneal HOAs, and objective optical quality, were conducted before surgery and at 1 day, 1 week, 1 month, and 3 months postoperatively.

### Statistical analysis

All statistical analyses were conducted using Statistical Package for the Social Sciences (SPSS) software (version 26.0; IBM Corporation, USA). The normality of the data was confirmed using the Shapiro-Wilk test. Categorical variables were presented as frequencies and percentages, while continuous variables were expressed as mean ± standard deviation. The chi-squared test was used for comparing sex, pre-, and postoperative visual acuity. A two-way repeated analysis of variance (ANOVA) measure, followed by the least significant difference (LSD) *t*-test, was used to compare preoperative and postoperative corneal aberrations. The age, refraction, and data on optical quality were not suitable for ANOVA analysis, so we employed Friedman's rank test for k-correlated samples. The preoperative and postoperative corneal HOAs were analyzed by Zernike polynomials. It is mainly the magnitude of the aberration affects the optical quality (21), when comparing the results, the absolute size of the aberration results was mainly considered to influence the optical quality. Correlation analysis was conducted using Spearman's method, with statistical significance set at *P* < 0.05.

## Results

### Baseline comparison

In the present study, a total of 186 eyes of 96 patients with moderate-to-high myopia who underwent refractive surgeries, including SMILE (98 eyes of 51 patients) and FS-LASIK (88 eyes of 45 patients), were recruited. Table 1 shows the demographic details and preoperative baseline characteristics of the patients. No statistically significant differences were observed between the two groups (*P* > 0.05). All surgeries were successfully performed without any observed postoperative complications.

**Table 1:** Demographic data and characteristics of patients.

**Parameters**	**SMILE (*n =* 51)**	**FS-LASIK (*n =* 45)**	**Z/*t***	***P***
Age (year)	25.64 ± 5.13	26.00 ± 5.39	−0.178	0.859
Sex (male/female)	14/37	12/33	0.394	0.530
S (D)	−5.73 ± 1.42	−5.74 ± 1.68	−0.055	0.956
C (D)	−0.56 ± 0.40	−0.61 ± 0.51	−0.536	0.592
SE (D)	−5.97 ± 1.43	−6.01 ± 1.76	−0.046	0.963
tHOA (μm)	0.40 ± 0.08	0.41 ± 0.09	−0.934	0.352
Piston (μm)	1.28 ± 0.50	1.39 ± 0.61	−1.336	0.183
Vertical tilt (μm)	−0.22 ± 0.42	−0.23 ± 0.4	0.017	0.987
Horizontal tilt (μm)	0.01 ± 0.38	0.04 ± 0.41	−0.468	0.640
Vertical trefoil(μm)	−0.02 ± 0.10	−0.01 ± 0.11	−0.893	0.373
Vertical coma (μm)	−0.10 ± 0.16	−0.11 ± 0.15	0.619	0.537
Horizontal coma (μm)	0.02 ± 0.14	0.03 ± 0.16	−0.425	0.671
Horizontal trefoil (μm)	−0.01 ± 0.10	0.00 ± 0.08	−0.885	0.377
Spherical aberration (μm)	0.27 ± 0.06	0.28 ± 0.08	−1.005	0.316
OSI	0.56 ± 0.28	0.63 ± 0.26	−1.952	0.051
MTF cutoff (c/deg)	41.28 ± 7.96	41.29 ± 7.16	−0.139	0.889
SR	0.24 ± 0.06	0.23 ± 0.05	−1.196	0.232

Data are shown as mean ± SD (range). SMILE, small incision lenticule extraction; FS-LASIK, femtosecond laser-assisted *in situ* keratomileusis; S, spherical; C, cylinder; SE, spherical equivalent refraction; tHOA, total higher-order aberration; OSI, object scatter index; MTF cutoff, modulation transfer function cutoff; SR, Strehl ratio.

### Visual acuity outcomes

**Figure 1:**
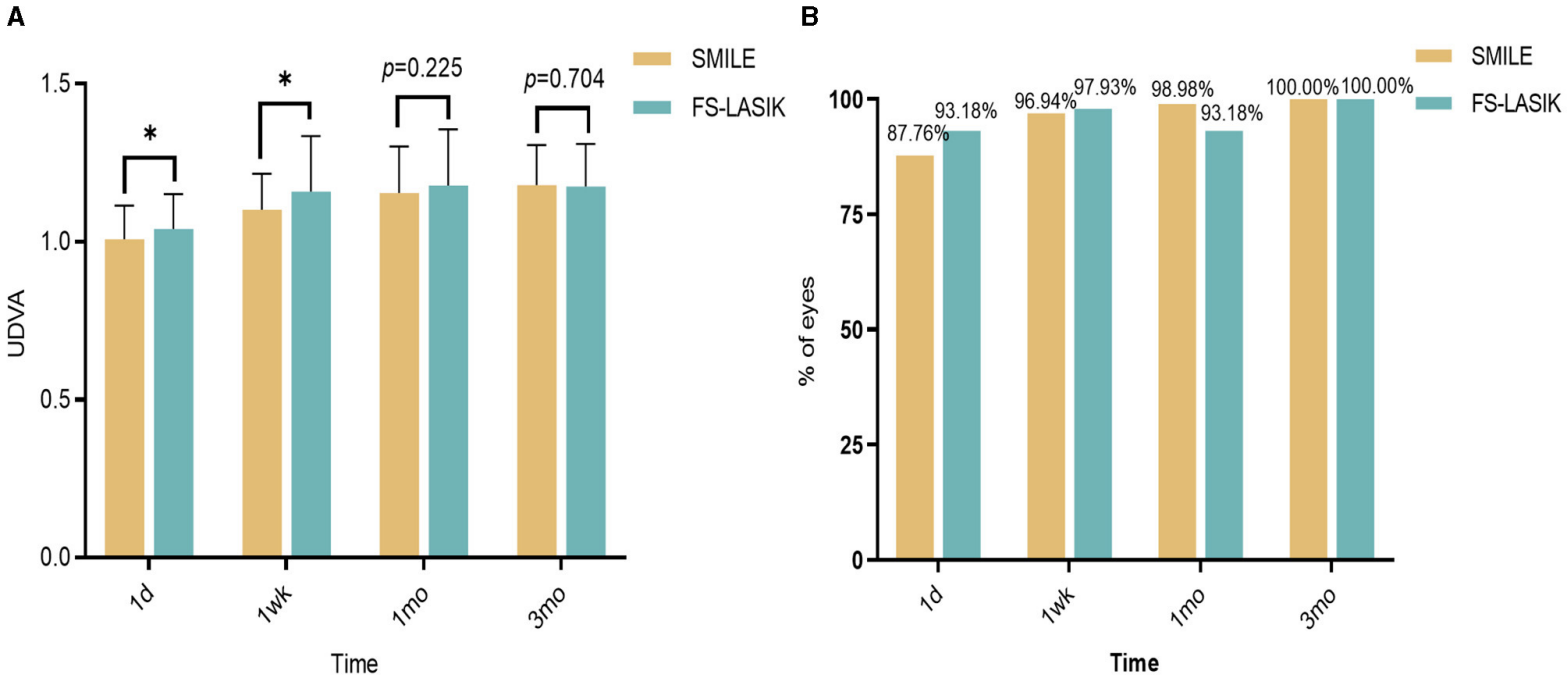
Changes in visual acuity after SMILE and FS-LASIK groups. **(A)** Changes in visual acuity after SMILE and FS-LASIK groups. **(B)** Comparison of the proportion of UDVA ≥ 1.0 in the SMILE group and the FS-LASIK group at different periods postoperative. *P < 0.05, statistically significant; UDVA, uncorrected distance visual acuity; SMILE, small incision lenticule extraction; FS-LASIK, femtosecond laser-assisted *in situ* keratomileusis.

There was a significant difference in the UDVA between the SMILE and FS-LASIK groups at 1 day and 1 week postoperatively (χ^2^ = −2.043, −2.475, *P* < 0.05), but there was no significant difference at 1 month and 3 months postoperatively (χ^2^ = −1.214, −0.380, *P* > 0.05; Figure 1A). The proportions of UDVA≥1.0 in the SMILE and FS-LASIK groups were 87.76% and 93.18% at 1 day postoperatively, 96.94% and 97.93% at 1 week postoperatively, 98.98% and 93.18% at 1 month postoperatively, and 100.00% and 100.00% at 3 months postoperatively, respectively. The proportions of UDVA ≥ 1.0 were slightly higher in the FS-LASIK group than in the SMILE group at 1 day and 1 week postoperatively. However, the proportions of UDVA ≥ 1.0 were slightly lower in the FS-LASIK group than in the SMILE group at 1 month and 3 months postoperatively. There was no statistically significant difference (*P* = 0.211, 1.000, 0.091, 0.797; Figure 1B).

### Refractive outcomes

**Figure 2:**
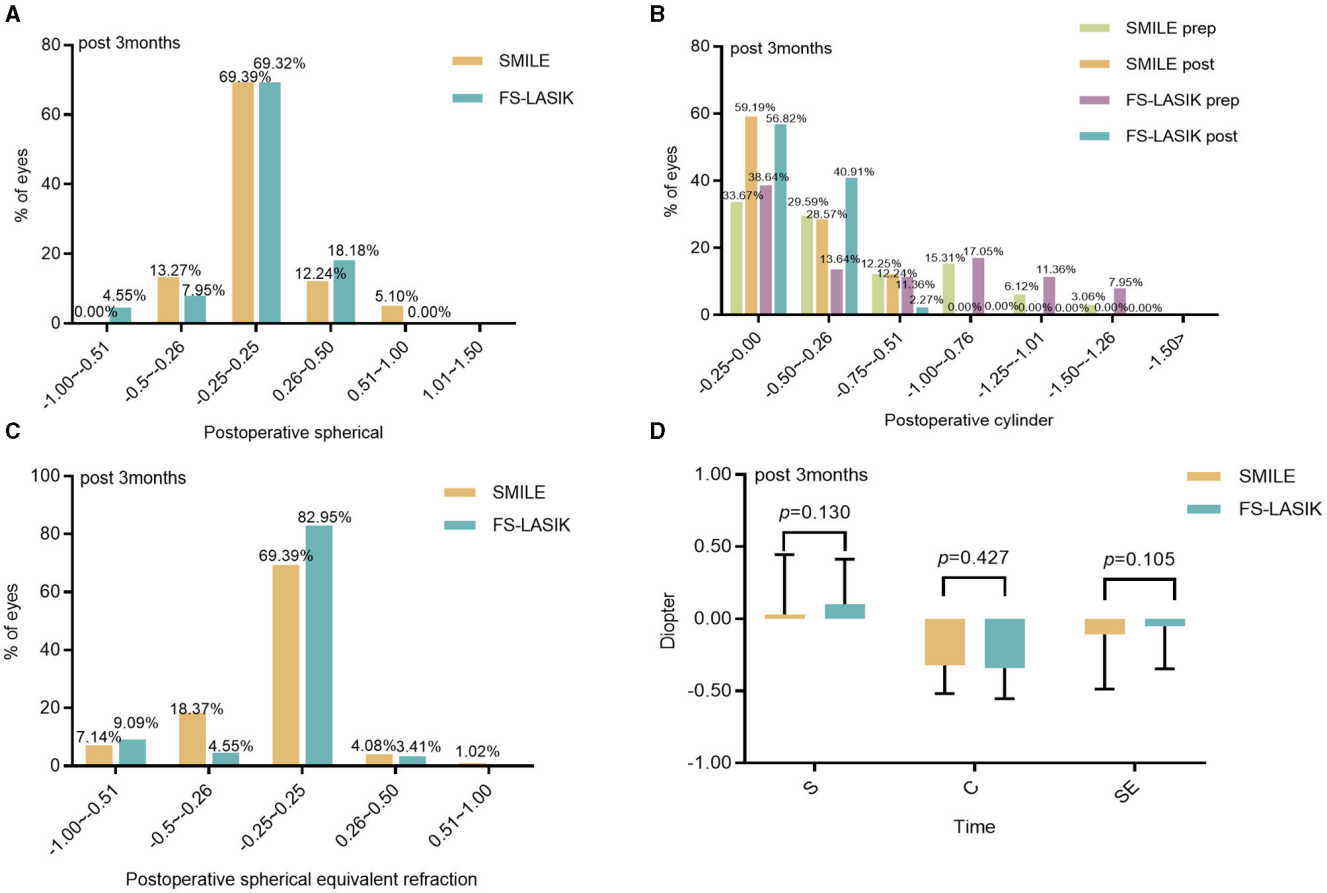
Refractive diopter after the operation between the SMILE and FS-LASIK. **(A)** Spherical; **(B)** cylinder; **(C)** spherical equivalent refraction; **(D)** refraction in the SMILE and FS-LASIK groups. SMILE, small incision lenticule extraction; FS-LASIK, femtosecond laser-assisted *in situ* keratomileusis; prep, preoperative; post, postoperative.

In 94.90% of SMILE patients and 95.45% of FS-LASIK patients, the spherical refraction was within ±0.50 D, and the spherical equivalent refraction was within ±0.50 D at 3 months post-procedure in 91.84% of SMILE patients and 90.91% of FS-LASIK patients. The difference between the two groups was not statistically significant (*Z* = −1.514, −1.620, *P* > 0.05; Figures 2A, C, D). At 3 months postoperatively, cylinders in the SMILE and FS-LASIK groups were (−0.36 ± 0.21) D and (−0.32 ± 0.19) D, respectively. The postoperative cylinder was reduced in both groups compared to the preoperative measurement, and there was no significant difference between the two groups (*P* = 0.427; Figures 2B, D).

### Corneal aberrations

**Figure 3:**
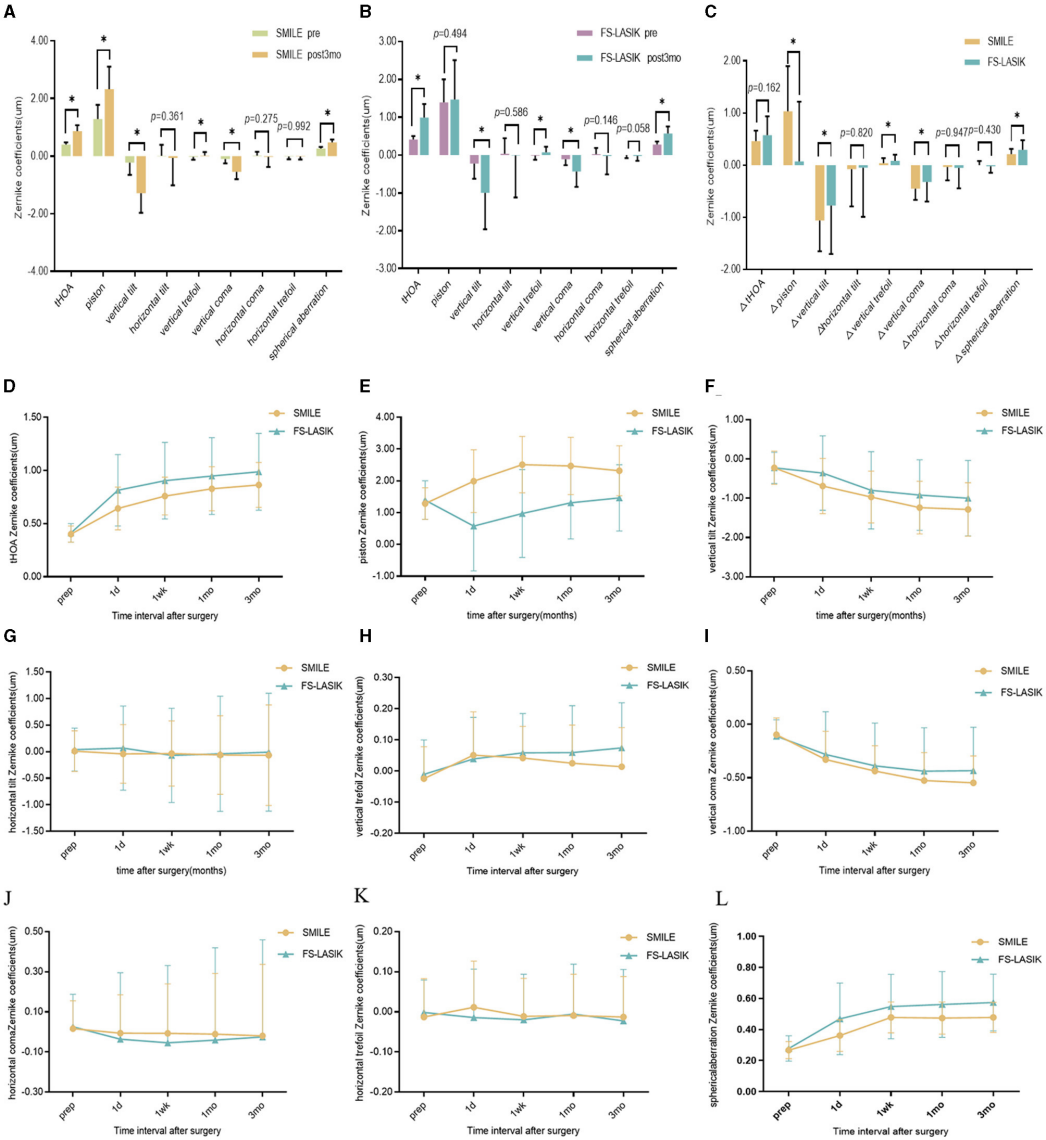
Corneal aberrations in the SMILE and FS-LASIK groups. **(A)** Mean of individual Zernike coefficient in the SMILE group; **(B)** mean of individual Zernike coefficient in the FS-LASIK group; **(C)** variation of each Zernike coefficient in corneal aberrations; **(D)** preoperative and postoperative root mean square of tHOA; **(E)** preoperative and postoperative of Zernike coefficient in piston; **(F)** preoperative and postoperative of Zernike coefficient in vertical tilt; **(G)** preoperative and postoperative of Zernike coefficient in horizontal tilt; **(H)** preoperative and postoperative of Zernike coefficient in vertical trefoil; **(I)** preoperative and postoperative of Zernike coefficient in vertical coma; **(J)** preoperative and postoperative of Zernike coefficient in horizontal coma; **(K)** preoperative and postoperative of Zernike coefficient in horizontal trefoil; **(L)** preoperative and postoperative of Zernike coefficient in spherical aberration. SMILE, small incision lenticule extraction; FS-LASIK, femtosecond laser-assisted *in situ* keratomileusis; prep, preoperative; post, postoperative; tHOA, total higher-order aberration; 1d, 1 day; 1wk, 1 week; 1mo, 1 month; 3mo, 3 months. *P < 0.05, statistically significant.

The absolute magnitude of tHOA, vertical tilt, vertical coma, and spherical aberration (SA) increased at 1 day, 1 week, 1 month, and 3 months postoperatively in the SMILE and FS-LASIK groups (*P* < 0.05; Table 2; Figures 3A, B, D, F, I, L). There was no significant difference in horizontal tilt compared with preoperative levels in both groups. The piston was increased postoperatively at each point of time in the SMILE group, and the piston decreased at 1 day postoperatively and gradually increased at 1 week in the FS-LASIK group. The absolute magnitude of the vertical trefoil was statistically significant when compared to the preoperative data in both groups (*P* < 0.05). There were no significant differences in the SMILE group compared to preoperative horizontal coma at 1 day, 1 week, 1 month, and 3 months (*P* > 0.05). At 1 day, 1 week, and 1 month postoperatively, the horizontal coma in the FS-LASIK group was statistically significant compared to preoperative data (*P* < 0.05). Compared to the horizontal trefoil before surgery, there was statistical significance at 1 day postoperatively in the SMILE group and at 1 week postoperatively in the FS-LASIK group (*P* < 0.05; Table 2). The corneal aberrations were essentially stabilized at 1 month postoperatively (Post 1 month vs. Post 3 month: *P* > 0.05); however, there were still some fluctuations observed (Figures 3D–L).

**Table 2:** Comparison of corneal higher-order aberration parameters between the two groups.

**Group**	**Prep**	**1 day**	**1 week**	**1 month**	**3 months**	***P* (prep. vs. 3 months)**
**tHOA**						
SMLIE	0.40 ± 0.08	0.64 ± 0.20^a^	0.76 ± 0.18^a^	0.83 ± 0.21^a^	0.86 ± 0.21	*P <* 0.05
FS-LASIK	0.41 ± 0.09	0.81 ± 0.34^a^	0.90 ± 0.36^a^	0.95 ± 0.36^a^	0.99 ± 0.36	*P <* 0.05
*P*	0.352	*P <* 0.05	*P <* 0.05	0.105	0.050	
**Piston**						
SMLIE	1.28 ± 0.5	1.97 ± 1.02^a^	2.51 ± 0.89^a^	2.47 ± 0.90^a^	2.32 ± 0.79	*P <* 0.05
FS-LASIK	1.39 ± 0.61	0.58 ± 1.41^a^	0.97 ± 1.38^a^	1.31 ± 1.14	1.47 ± 1.04	*0.494*
*P*	0.183	*P <* 0.05	*P <* 0.05	*P <* 0.05	*P <* 0.05	
**Vertical tilt**						
SMLIE	−0.22 ± 0.42	−0.69 ± 0.7^a^	−0.97 ± 0.66^a^	−1.24 ± 0.67^a^	−1.29 ± 0.68	*P <* 0.05
FS-LASIK	−0.23 ± 0.4	−0.36 ± 0.95	−0.80 ± 0.98^a^	−0.92 ± 0.90^a^	−1.00 ± 0.96	*P <* 0.05
*P*	0.987	*P <* 0.05	0.165	*P <* 0.05	*P <* 0.05	
**Horizontal tilt**						
SMLIE	0.01 ± 0.38	−0.04 ± 0.55	−0.03 ± 0.61	−0.06 ± 0.74	−0.07 ± 0.95	*0.361*
FS-LASIK	0.04 ± 0.41	0.07 ± 0.79	−0.07 ± 0.89	−0.04 ± 1.08	−0.01 ± 1.11	*0.586*
*P*	0.640	0.268	0.749	0.872	0.713	
**Vertical trefoil**						
SMLIE	−0.02 ± 0.10	0.05 ± 0.14^a^	0.04 ± 0.10^a^	0.03 ± 0.12^a^	0.01 ± 0.13	*P <* 0.05
FS-LASIK	−0.01 ± 0.11	0.04 ± 0.13^a^	0.06 ± 0.13^a^	0.06 ± 0.15^a^	0.07 ± 0.15	*P <* 0.05
*P*	0.373	0.546	0.328	0.090	*P <* 0.05	
**Vertical coma**						
SMLIE	−0.10 ± 0.16	−0.33 ± 0.26^a^	−0.44 ± 0.24^a^	−0.53 ± 0.26^a^	−0.55 ± 0.25	*P <* 0.05
FS-LASIK	−0.11 ± 0.15	−0.28 ± 0.40^a^	−0.39 ± 0.40^a^	−0.43 ± 0.40^a^	−0.43 ± 0.41	*P <* 0.05
*P*	0.537	0.342	0.321	0.051	*P <* 0.05	
**Horizontal coma**						
SMLIE	0.02 ± 0.14	−0.01 ± 0.19	−0.01 ± 0.25	−0.01 ± 0.30	−0.02 ± 0.36	0.275
FS-LASIK	0.03 ± 0.16	−0.04 ± 0.33^a^	−0.05 ± 0.38^a^	−0.04 ± 0.46^a^	−0.03 ± 0.49	0.146
*P*	0.671	0.460	0.326	0.605	0.931	
**Horizontal trefoil**						
SMLIE	−0.01 ± 0.10	0.01 ± 0.11^a^	−0.01 ± 0.09	−0.01 ± 0.10	−0.01 ± 0.10	0.992
FS-LASIK	0.00 ± 0.08	−0.01 ± 0.12	−0.02 ± 0.11^a^	−0.01 ± 0.12	−0.02 ± 0.13	0.058
*P*	0.377	0.138	0.563	0.823	0.572	
**Spherical aberration**						
SMLIE	0.27 ± 0.06	0.36 ± 0.10^a^	0.48 ± 0.10^a^	0.47 ± 0.10^a^	0.48 ± 0.10	*P <* 0.05
FS-LASIK	0.28 ± 0.08	0.47 ± 0.23^a^	0.55 ± 0.21^a^	0.56 ± 0.21^a^	0.57 ± 0.18	*P <* 0.05
*P*	0.316	*P <* 0.05	*P <* 0.05	*P <* 0.05	*P <* 0.05	

Data are shown as mean ± SD; SMILE, small incision lenticule extraction; FS-LASIK, femtosecond laser-assisted *in situ* keratomileusis; tHOA, total higher-order aberration; Prep, preoperative. ^a^significant compared with preoperative values.

There were no significant differences in postoperative horizontal tilt, horizontal coma, and horizontal trefoil at each point in time between the two groups (*P* > 0.05). The SA in the FS-LASIK group was slightly higher than that in the SMILE group at 1 day, 1 week, 1 month, and 3 months postoperatively (*P* < 0.05). The piston has the opposite result: the piston in the SMILE group was higher than that in the FS-LASIK group (*P* < 0.05). At both 1 day and 1 week, the FS-LASIK group had a slightly higher tHOA than the SMILE group (*Z* = −3.645, −2.110, *P* < 0.05), but there was no significant difference at 1 month and 3 months (*P* > 0.05). At 3 months postoperatively, the absolute magnitude of the vertical trefoil was greater in the FS-LASIK group than in the SMILE group (*t* = −0.033, *P* < 0.05). Additionally, the absolute magnitude of the vertical coma was lower in the FS-LASIK group than in the SMILE group (*t* = −2.257, *P* < 0.05; Table 2). There were no significant differences in ΔtHOA, Δhorizontal tilt, Δhorizontal coma, and Δhorizontal trefoil between the SMILE and FS-LASIK groups (*P* > 0.05). The FS-LASIK group had a slightly higher Δvertical trefoil and ΔSA than the SMILE group (*Z* = −2.530, −3.295, respectively, *P* < 0.05), although the Δpiston, Δvertical tilt, and Δvertical coma was lower than that in the SMILE group (*t* = 6.502, −2544, *Z* = −3.163, *P* < 0.05; Figure 3C).

### Objective optical quality

**Figure 4:**
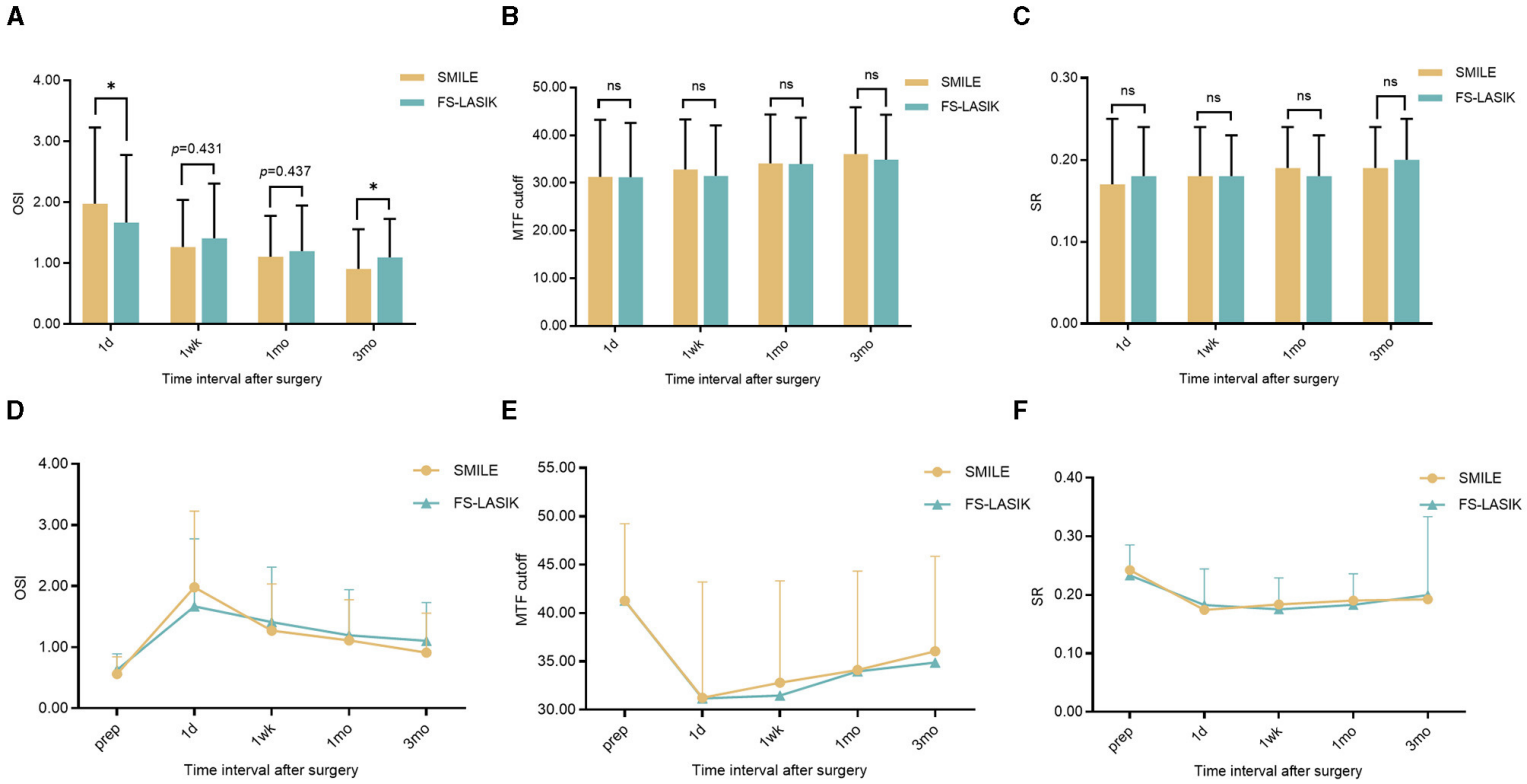
Visual quality in the SMILE and FS-LASIK groups. **(A)** Changes in OSI after the operation between the SMILE and FS-LASIK groups; **(B)** changes in MTF cutoff after the operation between the SMILE and FS-LASIK groups; **(C)** changes in SR after the operation between the SMILE and FS-LASIK groups; **(D)** preoperative and postoperative of OSI; **(E)** preoperative and postoperative of MTF cutoff; **(F)** preoperative and postoperative of SR. SMILE, small incision lenticule extraction; FS-LASIK, femtosecond laser-assisted *in situ* keratomileusis; OSI, objective scatter index; MTF cutoff, modulation transfer function cutoff; SR, Strehl ratio; prep, preoperative. ^ns^*P*, not significant; *P < 0.05, statistically significant.

At 1 day, 1 week, 1 month, and 3 months postoperatively, the OSI was significantly higher in both groups compared to the preoperative data; the MTF cutoff and SR were significantly lower than those before surgery in the SMILE and FS-LASIK groups, and the differences were statistically significant (*P* < 0.05). At 3 months postoperatively, measurements were still unstable in both groups (Table 3; Figures 4D–F).

**Table 3:** Comparison of the objective visual quality parameters between the two groups.

**Group**	**Prep**	**1 day**	**1 week**	**1 month**	**3 months**	***P* (prep. vs. 3 months)**
**OSI**						
SMLIE	0.56 ± 0.28	1.98 ± 1.25^a^	1.27 ± 0.77^a^	1.11 ± 0.67^a^	0.91 ± 0.65	*P <* 0.05
FS-LASIK	0.63 ± 0.26	1.67 ± 1.11^a^	1.41 ± 0.90^a^	1.20 ± 0.75^a^	1.10 ± 0.63	*P <* 0.05
*P*	0.051	*P <* 0.05	0.431	0.437	*P <* 0.05	
**MTF cutoff**						
SMLIE	41.28 ± 7.96	31.25 ± 11.98^a^	32.80 ± 10.53^a^	34.11 ± 10.23^a^	36.06 ± 9.80	*P <* 0.05
FS-LASIK	41.29 ± 7.16	31.19 ± 11.40^a^	31.46 ± 10.63^a^	33.97 ± 9.75^a^	34.89 ± 9.43	*P <* 0.05
*P*	0.889	0.948	0.462	0.977	0.302	
**SR**						
SMLIE	0.24 ± 0.06	0.17 ± 0.08^a^	0.18 ± 0.06^a^	0.19 ± 0.05^a^	0.19 ± 0.05	*P <* 0.05
FS-LASIK	0.23 ± 0.05	0.18 ± 0.06^a^	0.18 ± 0.05^a^	0.18 ± 0.05^a^	0.20 ± 0.13	*P <* 0.05
*P*	0.232	0.164	0.417	0.275	0.152	

Data are shown as mean ± SD; SMILE, small incision lenticule extraction; FS-LASIK, femtosecond laser-assisted *in situ* keratomileusis; OSI, Objective scatter index; MTF cutoff, modulation transfer function cutoff; SR, Strehl ratio; Prep, preoperative. ^a^significant compared with preoperative values.

There was a statistically significant difference in OSI between the two groups at 1 day and 3 months postoperatively (*Z* = −1.968, −2.843; *P* < 0.05; Table 3; Figure 4A), but there was no significant difference between the two groups at 1 week and 1 month postoperatively (*Z* = −0.788, −0.777, respectively; *P* > 0.05). Compared to the SMILE group, the postoperative MTF cutoff was slightly higher at each time in the FS-LASIK group; however, there was no statistical difference between the two groups (*P* > 0.05; Table 3; Figure 4B). In addition, there were no significant differences in SR between the two groups at 1 day, 1 week, 1 month, and 3 months postoperatively (*P* > 0.05; Table 3; Figure 4C).

### Correlation between corneal aberration and visual quality

**Figure 5:**
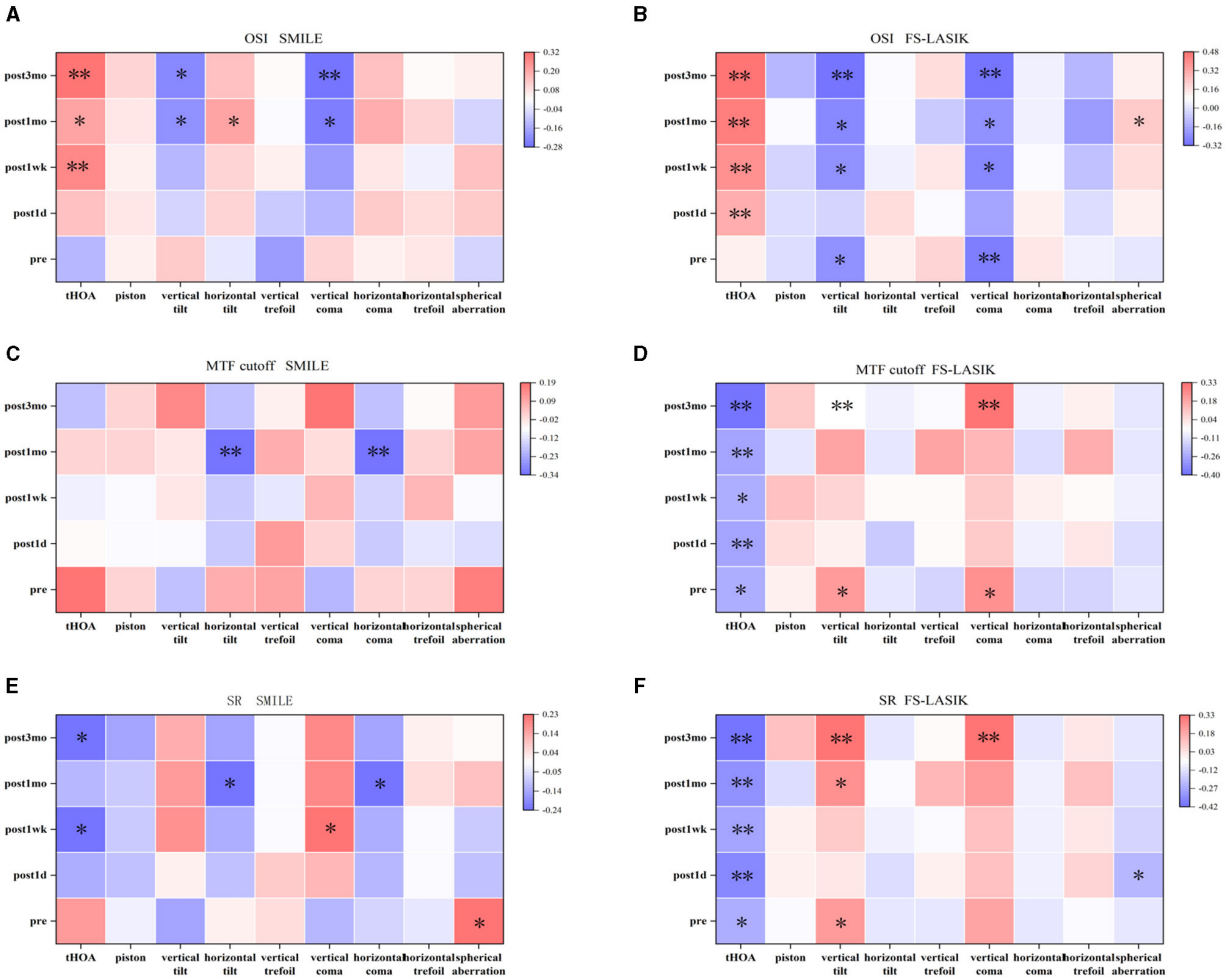
Correlation between corneal wavefront aberration and visual quality. **(A)** Correlation between corneal wavefront aberration and objective scatter index in SMILE. **(B)** Correlation between corneal wavefront aberration and objective scatter index in FS-LASIK. **(C)** Correlation between corneal wavefront aberration and modulation transfer function (MTF) cutoff in SMILE. **(D)** Correlation between corneal wavefront aberration and MTF cutoff in FS-LASIK. **(E)** Correlation between corneal wavefront aberration and Strehl ratio (SR) in SMILE. **(F)** Correlation between corneal wavefront aberration and SR in FS-LASIK. *P < 0.05. **P < 0.01.

Postoperative OSI was positively correlated with corneal tHOA (0.261 ≤ *R* ≤ 0.483, *P* < 0.05) and was negatively correlated with vertical tilt and vertical coma (−0.315 ≤ *R* ≤ −0.209, *P* < 0.05) in both groups (Figures 5A, B). Postoperative SR was negatively correlated with corneal tHOA (−0.417 ≤ *R* ≤ −0.232, *P* < 0.05) and was positively correlated with vertical coma (*R* = 0.223, 0.328, *P* < 0.05) in both groups (Figures 5E, F). Postoperative OSI was positively correlated with horizontal tilt (*R* = 0.207, *P* < 0.05); postoperative MTF cutoff and SR were negatively correlated with horizontal tilt and horizontal coma (−0.336 ≤ *R* ≤ −0.236, *P* < 0.05) in the SMILE group (Figures 5A, C, E). In the FS-LASIK group, postoperative MTF cutoff showed a negative correlation with tHOA (−0.403 ≤ *R* ≤ −0.236, *P* < 0.05), and postoperative SR exhibited a negative correlation with SA (*R* = −0.233, *P* < 0.05). Additionally, postoperative MTF cutoff and SR demonstrated positive correlations with vertical tilt and vertical coma (0.215 ≤ *R* ≤ 0.328, *P* < 0.05) in the FS-LASIK group (Figures 5D, F).

## Discussion

In the present research, both SMILE and FS-LASIK procedures demonstrated excellent safety, effectiveness, and predictability in correcting moderate-to-high myopia, aligning with the findings of earlier studies (22, 23). Both surgical procedures aim to correct refractive error by altering corneal curvature and morphology (24), but they may also lead to an increase in wavefront aberrations with varying degrees of optical quality loss in patients after surgery. Previous studies have found differences in postoperative visual quality at different periods of follow-up after both procedures (25, 26). Hence, the present study focused on the comparative analysis of corneal wavefront aberration and objective visual quality after SMILE and FS-LASIK with moderate-to-high myopia in the early postoperative period.

Visual acuity and refraction are commonly utilized to assess the effectiveness of corneal refractive surgery. Visual acuity can be significantly improved after surgery, according to our study. The proportions of UDVA ≥ 1.0 in the SMILE and FS-LASIK groups were 98.98% and 93.18% at 1 month postoperatively, and 100.00% and 100.00% at 3 months postoperatively, which is similar to the results of previous studies (27, 28). The present study indicated that the UDVA after FS-LASIK surpasses that of SMILE in 1 day and 1 week. A previous study (10) compared and analyzed the short-term postoperative UDVA between SMILE and FS-LASIK, it was found that UDVA was higher in the FS-LASIK group, indicating that this group had better UDVA at 1 week and 1 month post-surgery; however, the difference between the two groups was not significant. Other studies have also documented better visual acuity on the 1^st^ day or within the 1^st^ week following FS-LASIK compared to SMILE (11, 12). These studies suggested that FS-LASIK may provide better visual acuity within the 1^st^ month after surgery, potentially due to boundary irregularities and haze present during the early postoperative period in SMILE (29). Meanwhile, our results also indicated that the piston was higher in the early postoperative period following SMILE compared to the findings of FS-LASIK, suggesting a potentially prolonged corneal recovery in the early postoperative phase of SMILE.

In addition, recent studies on optimizing energy settings in low-energy SMILE have shown that early postoperative visual outcomes are comparable to those of FS-LASIK (30, 31). Ji et al. (30) compared the visual acuity recovery within 3 months after SMILE with a high-laser energy setting (115–150 nJ) and a low-laser energy setting (110 nJ) and found that a low-laser energy setting leads to better visual acuity recovery within 1 month; Hamilton et al. (31) found that the effect of visual acuity recovery in the early stage of the postoperative period was the same as that of FS-LASIK; Varman et al. (32) used a low-laser energy setting of 110 nJ in SMILE, and all patients achieved UDVA 20/20 as early as postoperative 1 day without intraoperative opaque bubble layers or difficult dissection. They suggested that the energy setting in SMILE might influence the visual recovery outcomes in the early postoperative period. In this study, a high-laser energy setting of 130 nJ was utilized intraoperatively in the SMILE group. Concurrently, a higher piston was observed in the SMILE group compared to the FS-LASIK group at 1 day and 1 week postoperatively. This suggests that the high-laser energy setting may influence patients' postoperative corneal recovery, potentially affecting early visual acuity.

Our study found that postoperative manifest refraction was similar between the SMILE and FS-LASIK at 1 day, 1 week, 1 month, and 3 months, which was consistent with previous studies (7, 33, 34) as well as with the findings of the present study. The results showed that both groups were effective in correcting moderate-to-high myopia; no patients suffered complications such as high intraocular pressure and haze after surgery, and the postoperative visual acuity recovered better.

Wavefront aberration is divided into higher-order and lower-order aberrations, and the absolute magnitude of higher-order aberration has a greater impact on visual quality (21). The OPD-Scan III is a multifunctional device that can measure total, corneal, and intraocular higher-order aberrations within 30 s. Wavefront aberrations are calculated using automatic retinoscopy (dynamic skiascopy) over a 9.5-mm pupil zone, utilizing up to 2,520 points. This method is characterized by its short measurement time and wide measurement range. It has good accuracy and reproducibility in measuring ocular wavefront aberrations (35). The piston measurement quantifies the degree of corneal impact of various surgical procedures and helps the clinician evaluate the patient's postoperative corneal recovery. Therefore, we mainly used the OPD-Scan III to measure the corneal aberration before and after the patient's surgery. Additionally, we conducted a further analysis of the effect of wavefront aberration on objective optical quality.

Our study found that corneal tHOA, vertical tilt, vertical trefoil, vertical coma, and SA at 3 months were higher than preoperatively in both groups. Previous studies have also found that trefoil, coma, and SA increase after refractive surgery (36–38). There are a number of reasons that may lead to increased corneal aberration postoperatively, such as off-center, irregular cuts, an unsmooth corneal stromal bed during flap making, unskilled handling during lens removal, the size of the corneal incision, postoperative corneal recovery, and other corneal biological characteristics. Additionally, postoperative tHOA and SA were slightly higher in the FS-LASIK group than in the SMILE group. The cutting of nerve endings during corneal flap creation in FS-LASIK can degrade tear film quality, potentially impacting corneal aberrations. Additionally, even minimal displacement of the corneal flap in FS-LASIK may significantly influence wavefront aberrations. Meanwhile, SMILE causes less surgical trauma and can maximize the protection of corneal biomechanical properties and integrity, which effectively avoids flap-induced aberration variations and further improves the recovery of patients' postoperative optical quality. Another reason may be that SMILE is equipped with a reduced SA program design during the cutting process, which results in smaller SA changes in the postoperative period (33). However, our study found that SMILE induced more vertical tilt and vertical coma compared to FS-LASIK. Several possible reasons that might explain this result are as follows: (1) FS-LASIK requires the creation of a corneal flap (39), which has essentially the same impact on peripheral corneal forces and biomechanical effects, whereas most of the incisions in SMILE are located above the cornea, and their incision direction and forces may induce more vertical tilt and vertical coma in the operative period. (2) SMILE does not incorporate iris registration technology, which compensates for pupillary cyclotorsion and offsets to ensure proper centration (40). (3) Compared to the transition zone around the corneal flap in FS-LASIK, the vertical edge of the refractive lenticule in SMILE might induce more vertical tilt and vertical coma in the postoperative period. Our study showed that the measurements of wavefront aberration were essentially stabilized at 1 month postoperatively (Post 1 month vs. Post 3 month, *P* > 0.05), but still fluctuated. Zheng et al. (41) found that corneal higher-order aberrations and optical quality of FS-LASIK, WF-LASIK, and SMILE were stable at 3 months after surgery, and that corneal higher-order aberrations showed a decreasing trend at 12 months compared with 6 months after surgery, which suggested that the cornea was still in the repair period at 3 months postoperatively and that the patients should pay attention to ocular hygiene and use reasonable medications.

Generally, common methods for evaluating visual quality include both subjective and objective assessments. Subjective visual quality evaluation has a certain degree of subjectivity, as it depends on the patient's cognitive understanding and level of cooperation. Objective visual quality assessment minimizes errors caused by patient cooperation and has significant clinical application value. Our study utilized the OQAS II analyzer, based on the double-pass technique, which is widely used for objective optical quality inspection and has demonstrated reliable repeatability in previous research (42, 43). The results of our study demonstrated a consistent decline in optical quality over a 3-month period following both SMILE and FS-LASIK procedures. No notable differences were observed in MTF cutoff and SR between SMILE and FS-LASIK. The OSI for SMILE was higher than that for FS-LASIK at 1 day postoperatively but was lower than that for FS-LASIK at 3 months postoperatively. This finding was different from some previous observations regarding SMILE and FS-LASIK surgery (44–46). Qin et al. (44) found no statistically significant difference in SR and MTF cutoff at any time point postoperatively compared with before surgery for high myopia correction with SMILE. Miao et al. (45) employed the OQAS II to assess the optical quality following SMILE and observed that the optical quality remained comparable to that observed before surgery. Wu et al. (46) reported the MTF cutoff and SR showed no significant change compared to preoperative levels. While there was an increase in OSI at 1 month postoperatively; it subsequently declined to preoperative levels at 6 months. It may be associated with the patient's refractive error, the optical quality of the examination equipment, the measurement environment, the surgical equipment and design, and the patient's ocular factors. The present study showed that early postoperative optical quality was lower than preoperative optical quality. Postoperative corneal tHOA, vertical tilt, and vertical coma were correlated with objective visual quality, and the objective visual quality of FS-LASIK is greatly affected by corneal aberrations. Previous studies have suggested that coma may have a protective effect on postoperative visual quality (13). In this study, we observed a weak correlation between the absolute magnitude of vertical coma and objective visual quality in both groups. However, this correlation was minor. Vertical coma induces a comet-like “trailing” effect, which is recognized as one of the most detrimental higher-order aberrations to image perception (47, 48).

In addition, this study has some limitations. First, it focused solely on objective visual quality. To provide a more comprehensive evaluation of postoperative visual quality, future research should also include subjective visual quality assessments. Second, our study did not focus on the optical quality at different pupil diameters. Third, the patients had moderate-to-high myopia, making it impractical to exclude the influence of adjustment factors and refraction on the results. We anticipate further research examining the optical quality following various corneal refractive procedures in patients with different degrees of myopia. Additional research is necessary to extend the follow-up period and increase the number of patients to more thoroughly assess objective optical quality in individuals with moderate-to-high myopia after undergoing SMILE and FS-LASIK procedures.

## Conclusion

Both SMILE and FS-LASIK could be effective in improving visual acuity in patients with moderate-to-high myopia. Postoperatively, both procedures showed increased corneal HOAs and delayed optical quality. Although SMILE induces more vertical tilt and vertical coma, and the cornea may require a longer recovery period than compared with FS-LASIK, SMILE also possesses a better ability to reduce the induction of tHOA and SA. Additionally, OPD-Scan III combined with OQAS II is a useful supplementary inspection for assessing the optical quality following refractive surgery.

## Data Availability

The original contributions presented in the study are included in the article/supplementary material, further inquiries can be directed to the corresponding author.
